# Pancreas-on-a-Chip Technology for Transplantation Applications

**DOI:** 10.1007/s11892-020-01357-1

**Published:** 2020-11-18

**Authors:** Shadab Abadpour, Aleksandra Aizenshtadt, Petter Angell Olsen, Kayoko Shoji, Steven Ray Wilson, Stefan Krauss, Hanne Scholz

**Affiliations:** 1 Department of Transplant Medicine and Institute for Surgical Research, Oslo University Hospital, Post Box 4950, Nydalen, N-0424 Oslo, Norway; 2 Hybrid Technology Hub-Centre of Excellence, Institute of Basic Medical Sciences, University of Oslo, Oslo, Norway; 3 Department of Chemistry, University of Oslo, Oslo, Norway; 4 Institute of Immunology, Oslo University Hospital, Oslo, Norway

**Keywords:** Islet transplantation, Pancreas transplantation, Stem cell-derived beta-like cells, Type 1 diabetes, Organ-on-a-chip, Microfluidic systems, Pancreas-on-a-chip, Multi-organ-on-a-chip

## Abstract

**Purpose of Review:**

Human pancreas-on-a-chip (PoC) technology is quickly advancing as a platform for complex in vitro modeling of islet physiology. This review summarizes the current progress and evaluates the possibility of using this technology for clinical islet transplantation.

**Recent Findings:**

PoC microfluidic platforms have mainly shown proof of principle for long-term culturing of islets to study islet function in a standardized format. Advancement in microfluidic design by using imaging-compatible biomaterials and biosensor technology might provide a novel future tool for predicting islet transplantation outcome. Progress in combining islets with other tissue types gives a possibility to study diabetic interventions in a minimal equivalent in vitro environment.

**Summary:**

Although the field of PoC is still in its infancy, considerable progress in the development of functional systems has brought the technology on the verge of a general applicable tool that may be used to study islet quality and to replace animal testing in the development of diabetes interventions.

## Introduction

Type 1 diabetes (T1D), which is mainly referred to as the insulin-dependent disease, results from an immune-mediated destruction of beta cells within the pancreatic islets [1]. Exogenous insulin therapy has shown to be effective to maintain the glucose homeostasis and reduce the complications associated with T1D, but it poorly mimics the normal beta cell function and comes with the risk of hypoglycemic episodes [2]. Beta cell replacement therapy in the form of both pancreas transplantation and allogeneic islet transplantation has proven to be a safe alternative treatment to external insulin administration, offering more precise control over blood glucose, reducing incidents of unaware hypoglycemia, and improving quality of life in patients with T1D [3]. The indications for beta cell therapy and factors influencing the chosen strategy are not covered by this review and can be read elsewhere [4]. For pancreas and islet transplantations, the procurement of the donor organ is an essential upstream step for the success of the islet isolation procedure and for graft survival [5]. Advances of organ preservation using ex vivo machine perfusion technologies show that organs can be kept viable outside the organisms for extended time period and contribute to achieve better viability and improvement in graft function [6–8].

For islet transplantation, existing procedures involve isolation and purification of the islets from the rest of the pancreatic tissue which often leads to ischemic damage and a pro-inflammatory signature [9]. Prior to transplantation, islet quality is evaluated mainly by measurements of number of islets, sterility, purity, and viability. Depending on the release criteria at different centers, this also includes potency assays such as static or dynamic glucose-stimulated insulin secretion followed by a calculation of the stimulation index (SI). Commonly, SI > 1 is considered as enough evidence for a sufficient response of the isolated islets to blood glucose [10–12]. Unfortunately, this test does not necessarily correlate with the clinical outcome after islet transplantation, probably due to a variability of in vitro environmental factors [13].

Due to the limitations of suitable immune-matching donor tissue in terms of quantity and quality, as well as time and geographic limitations between the availability of donor material and acceptor readiness, alternatives are being explored. The last decade has mobilized significant resources for developing an unlimited source of insulin-producing cells that can be used for transplantation [14]. Strategies have primarily been based on attempts to glucose responsive cells through differentiation protocols starting from embryonic stem cells (ESCs) or pluripotent stem cells (PSCs) [15].

Immune compatibility is a central issue in all transplantation regimes. Although strategies such as encapsulation of islets or stem cell-derived beta-like cells enable the transplantation of cells without the need for immune suppression [16], these strategies are still under development. There has also been evidence showing that even autologous induced pluripotent stem cell (iPSC) differentiation and transplantation to patients could create immune responses in recipients [17]. Therefore, the use of immunosuppressive medications is an inevitable step [18, 19]. Establishment of the best available immunosuppressive regimen is challenging mainly due to the direct toxicity of immunosuppressive medications on islet viability and glucose responsiveness [20, 21].

While in vitro differentiation of stem cells to beta-like cells is slowly improving, awareness about the importance of the micro-environment of the beta cells and its standardized supply of oxygen, nutrition, and other factors is raising. Hence, platforms that provide a controlled micro-environment and enable rigid standardization of surrounding conditions are being developed.

Pancreas-on-a-chip (PoC), which refers mainly to the study of endocrine part of the pancreas on microfluidic chip, may be used as a standardized and real-time assessment platform for evaluating islet potency and quality. Organ-on-a-chip (OoC) technology, in general, promises to provide a test and interrogation environment that might replace the complexity of an animal model. OoC is a microfluidic-based device enabling to culture and grow living cells and organoid substructures in a controlled micro-environment. Commonly, OoC platforms recapitulate one or more aspects of the organ’s dynamics, functionality, and in vivo (patho) physiological responses under real-time monitoring of different cultured tissue types [22]. The OoC technology has been developed, aiming for better prediction of the preclinical drug testing and reduction of the use of animal for the pharmaceutical industry [23, 24].

One of the first papers on using cells in microfluidic platform was published in 1991, reporting a construction of a ventricular myocardium on a microfluidic system, which enables the first biophysical analysis of heart in vitro [25]. The first microfluidic platform to study the interaction of lung and liver tissue was introduced in 2004 [26•]. Since then, a range of microfluidics have been developed, mimicking diverse biological function of various tissue types including muscles, bones, liver, lung, brain, gut, and kidney [27].

OoC-based systems have the potential to be used for a range of applications such as personalized organ function and dysfunction, as well as pharmacological interventions that are particularly difficult to study in an isolated 2D or 3D in vitro laboratory setting. PoC devices that enable in a minimal equivalent environment to mimic the function of islets in particular may enable us to gain new information on the islet physiology and the impact of therapeutic interventions. Importantly, PoC technology also promises to advance standardization and quality control of the islets or stem cell-derived beta-like cells per se for replacement therapy. In this review, we will present the various developments toward PoC platforms and discuss the use of this technology for clinical transplantation.

## Pancreas-on-a-Chip Designs

The design of the chip depends largely on the function of the organ. Therefore, each organ type needs individualized system to mimic its micro-environment. Microfluidic platforms for islets may have trapping sites in which islets are immobilized and cultured under medium flow [28•]. Trapping of islets is mostly done by micro-wells [29•, 30, 31, 32•, 33–35] or by constrictions [36••, 37–40] that are fabricated in micro-channels under continuous flow. Using such microfluidic platforms, islet viability and composition as well as hormone and metabolite secretions by islets could be tested “on-chip”. Perfusion is essential to maintain the viability of trapped islets, removal of metabolic products, and stimulation of islets with chemicals, hormones, and nutrients such as glucose. Precise fluid control is crucial to minimize any damage to the trapped islets and to analyze hormone kinetics in relation to biochemical and metabolic stimulation in the islets. Jun et al. have developed a microfluidic perfusion system providing an osmosis-driven low-speed flow (1.54–5.04 μm/s) comparable to in vivo interstitial flow levels. This allows culturing re-aggregated islet in “concave” micro-wells for a month with low flow rate that could prevent shear force damage to cells and provide continuous oxygen and nutrient supplies for islets. This model aids studying islet function post-isolation and has a potential to be used as an in vitro model for diabetic drug testing [29••]. Physical protection of islets from flow within a chip platform by placing islets in a micromesh sheet has also been reported as a strategy to avoid damaging of islets by shear force while allowing perfusion of oxygen and nutrient to the islet in the chip [41].

Size heterogeneity of isolated islets (50–400 μm) reduces the viability of islets in microfluidic chip due to the fact that large islets could damage in a greater extent by the flow rate. Therefore, having islets with different sizes in microfluidic platform could create challenges in the amount of oxygen and nutrient that islets need [42]. It has been shown that smaller islets perform better than large islets both in clinical settings [43] and in culture [44]. Standardization of islet size by engineering size-controlled cell clusters is one strategy to overcome the variation in oxygen and nutrient demand by islets in microfluidic platform and also flow rate damage in the system [45].

## Pancreas-on-a-Chip Application for Islet Quality Control Post-Isolation

**Fig. 1:**
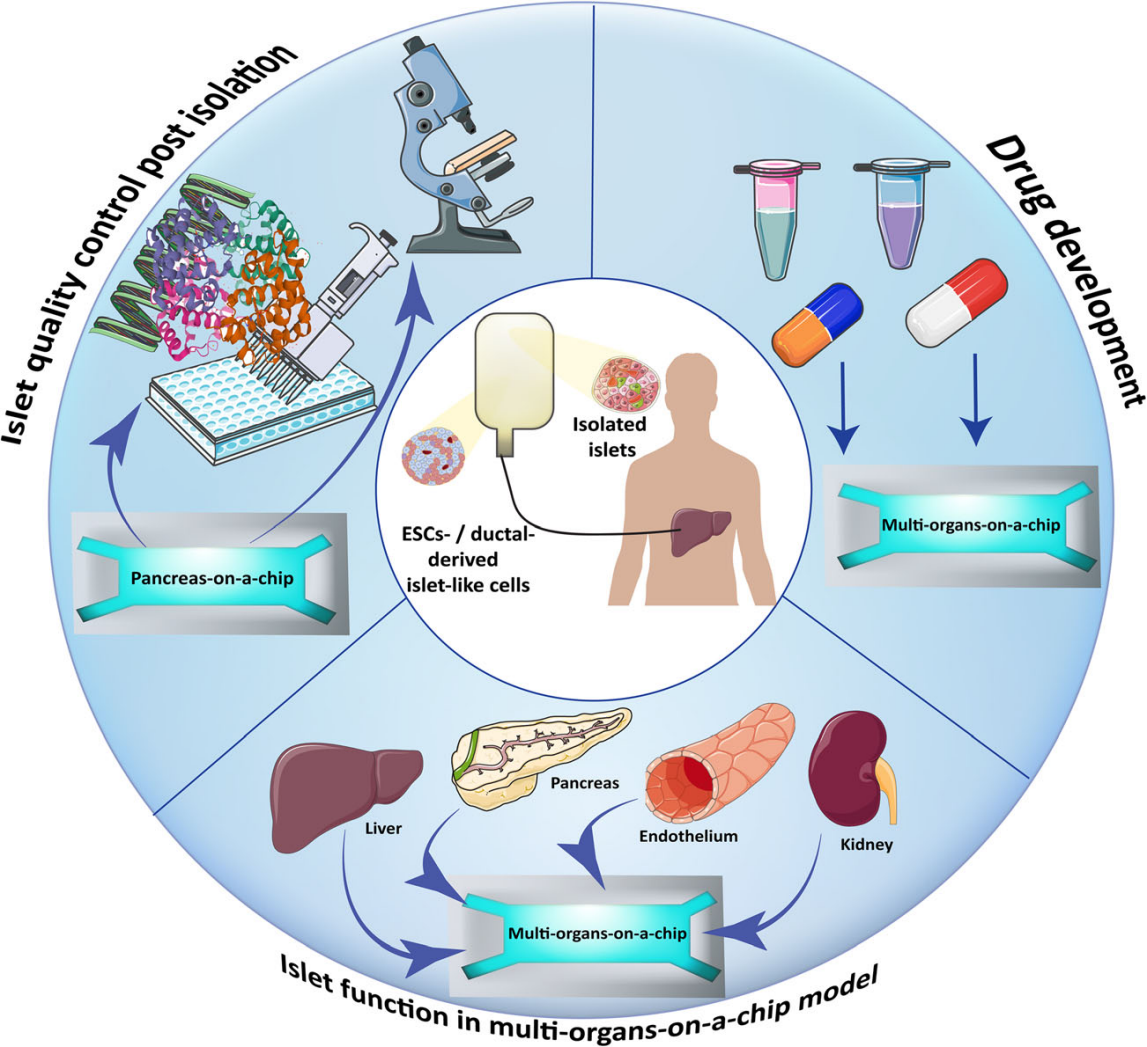
Pancreas-on-a-chip application for diabetes and islet transplantation research. Pancreas-on-a-chip platforms not only could be used as a system to perform islet quality control post-isolation procedure, but also can be used to investigate stem cell-derived beta-like cell function and compare them with primary islets in a standardized platform. Islet quality control in a standardized microfluidic platform could give one more comprehensive understanding of the isolated islet quality and function and predict outcome of the islet transplantation. Combining islets or stem cell-derived beta-like cells on chip with other organoids for example liver, kidney, or endothelial tissue not only increases our knowledge on islet function and their interactions with other tissue types, but also could be used as a valuable system for drug development including diabetes-related and immunosuppressive medications

Islet viability could be adversely affected by numerous factors during organ procurement and isolation procedure. These negative events increase the number of required islets post-transplantation in order to achieve insulin independence. Having reliable methods to study islet potency post-isolation could attribute to better evaluation of islet quality post-isolation (Fig. 1). Currently, an in vitro glucose-stimulated insulin secretion assay is widely used for assessing islet quality post-isolation procedure, and the results of this assay are considered as exclusion criteria for clinical islet transplantation in type 1 diabetic patients. However, it has been reported that poor insulin release in response to glucose in human islets does not necessarily predict the in vivo function of islet post-transplantation [46], and this method alone could not be used for studying islet potency post-isolation.

Transplanting isolated islets to a diabetic mouse model is also considered as a method to assess islet potency in reversing blood glucose and diabetic condition. However, this is a retrospect manner as it takes more than 30 days to get the information about islet quality. Therefore, this method could not be used as a quality control for isolated islets prior to transplantation. In addition, other factors such as the time of performing in vivo glucose response test and blood glucose measurements are important. Most of the analyses for evaluating islet grafts are performed during day; however, rodents are nocturnal animals and are more active during night. This affects the transplanted islet graft response and the result of islet quality control [47].

Several research groups have been developing microfluidic devices for evaluating islet function including islet response to glucose and insulin secretion, intracellular Ca^2+^, islet membrane capacities, and oxygen consumption rate (Table 1) [53–56]. The first microfluidic device designed to be used for human islet evaluation post-isolation is a polydimethylsiloxane (PDMS)-based device in which 100 islets are infused into a device with 7 mm diameter [32•]. The device has wells on the bottom with 500 μm diameter for trapping and culturing islets. The device is perfused using peristaltic pumps for circulating the culture media and glucose solutions for performing dynamic glucose responsiveness and Ca^2+^ measurement tests [32•].

**Table 1:** Generated PoC platforms and possible application of these platform in islet research

Model	Material in chip	Purpose	Outcome	Ref
Human and mouse pancreatic islets	PDMS	Microfluidic platform to study isolated islet function by measuring insulin secretion in response to glucose and fluorescent imaging of the mitochondrial membrane potential and intracellular calcium.	First microfluidic perfusion chamber for islets that allow study of dynamic insulin secretion in response to glucose and intracellular calcium measurement upon glucose challenge.	[32•]
C57BL/6 mice islets	PDMS	Online and real-time analysis of islet membrane action potential using integrated biosensors to the PoC platform.	Using only few islets, the chip with integrated biosensors allow measurement of membrane action potentials in islets upon treatment with different glucose concentrations. This platform could be combined with insulin analysis test to give a better overview of islet health.	[48]
Sprague-Dawley rat islets	PDMS	Dynamic culturing of islet spheroids under interstitial flow condition to reduce shear cell damage in microfluidic chip.	Dynamic culture of islet spheroids enhanced islet function and maintained islet endothelial cells up to 30 days inside the chip. This system increased reconstitution of islet extracellular matrix. The proposed model has a potential to be used as platform for following islet function post-isolation and also to be used as an in vitro model for testing diabetic medications.	[29••]
MIN6 psuedo-islets and isolated mouse islets.	Borosilicate glass	Using a see-through material for simultaneous measurement of fluorescence and oxygen consumption rate in a PoC model.	The platform enabled measurement of oxygen consumption, NAD(P)H auto-fluorescence, cytosolic Ca^2+^ concentration, and insulin secretion by islets.	[49]
Pancreatic human islets	Polycarbonate	Generation of microfluidic chip for islets with automated islet loading, direct glucose stimulation chip, and integrated insulin detection assay inside the chip.	This microfluidic chip provides a synchronized glucose stimulation and continuous insulin detection using an on-chip immunoassay. Using this system, there is no need for sampling. The platform is see-though which gives the possibility for imaging the islets inside the chip. This platform is made of polycarbonate which does not have the negative impact of PDMS on cultured islets.	[36••]
iPS cells	PDMS	Multi-layer microfluidic platform for in situ differentiation of iPSCs to islet organoids.	Direct aggregation and differentiation of iPSCs to beta- and alpha-like cells. Cells expressed endocrine transcription markers. They were positive for insulin and c-peptide protein expressions and responsive to glucose stimulation.	[50]
EndoC-βH3 cells	PDMS	Generation of microfluidic chip with self-guided trapping sites for human pseudo-islets in order to monitor insulin secretion kinetics and study pseudo-islet functionality using Raman microscopy.	Real-time monitoring of islets using Raman microscopy as well as dynamic sampling for biphasic glucose-stimulated insulin response analysis. Raman microscopy not only allowed tracing islet glucose responsiveness, but also visualized molecule structures such as lipids and gave a possibility to study mitochondrial activity.	[28•]
C57BL6 mice islets	PDMS	Developing microfluidic chip with a range of flow rate in order to improve culture medium exchange inside the islets and increase survival of the endothelial cells with pancreatic tissue.	Adjusted flow rate inside the chip resulted in double increase in endothelial density in islets compared to the classical culture system. The islets showed improvement in glucose-stimulated Ca^2+^ response and insulin secretion.	[51]
Human pancreatic islets	PDMS	Interaction of human primary pancreatic islets and liver spheroids using a microfluidic chip.	Co-culturing islets and liver organoids in microfluidic chip maintains the tissue function up to 15 days. Functional coupling of islets and liver tissue demonstrated insulin release in response to glucose by islets and promoted glucose uptake by liver organoids. The model could be used to study the effect of various diabetic medications on glucose regulation.	[52•]

The most common methods for analyzing secreted insulin by islets, either in standard culture or in microfluidic platforms, are capillary electrophoresis-based immunoassays (CEIA) and intracellular Ca^2+^ oscillation monitoring [29••, 30, 31, 33, 34, 40, 57]. Lomasney et al. have applied CEIA in a glass chip to detect insulin and islet amyloid polypeptide [31]. The chip has also flow switching feature that enable switching the glucose concentration every 5 s. Another method for detection of insulin secreted by islets is Ca^2+^ oscillation, which could visualize the exocytosis of insulin using fluorescent dyes and allows measurement of secreted insulin in response to stimulated glucose instantly and hence could be used as a pre-evaluation method in post islet isolation [29••, 30, 32•, 35, 40]. One of the disadvantages with most of the microfluidic devices is the sample size. Requiring a large number of islets makes these devices not suitable for quality control of islets post-isolation. Microfluidic devices with integrated biosensors in the form of extracellular electrodes engulfed to plasma membrane have been developed in order to provide a possibility to study and detect islet function from few numbers of islets in chip platform [48]. These biosensors have the ability to detect action potential in response to different concentration of glucose [48]. Some devices offer single islet sensitivity devices, which minimize the number of islets required for post-isolation functionality assessment [34]. Some of these microfluidic platforms have integrated automated systems for samplings, which gives the possibility of performing dynamic glucose-stimulated insulin test and detection of both prominent first phase and sustainable pulsatile second phase of insulin secretion [58].

Despite advances in the design of microfluidic devices, traditional sampling and liquid handling followed by ELISA quantification are still predominant. Glieberman et al. developed a fully automated polycarbonate-based device with integrated “on chip” insulin immunoassay [36••]. Implementing the insulin immunoassay detection reduces the required time and cost of sampling and analyzing secreted insulin by islets, which could make this device scalable for large applications. In addition, the presented device has a high sensitivity in detection of insulin. Therefore, it needs only a few numbers of islets with the possibility to retrieve the islets after glucose stimulation test for further analysis. Such a device gives also a possibility to customize the ELISA assay implemented in the device in order to measure other physiologically relevant stimulus such as incretin and glucagon [36••].

Recently, studies have reported the assessment of islet quality using oxygen consumption rate (OCR) [59, 60]. Moreover, the calculation of ATP/ADP ratio in addition to OCR measurement and glucose-stimulated insulin secretion assay is considered as a method for evaluating islet potency [61]. Analysis of islet viability post-isolation using measurement of miRNAs in particular miRNA-375 and also studying the DNA methylation pattern on insulin promoter have been reported to correlate with the outcome of islet transplantation [62].

A recently published study reported a new microfluidic device in which islets are cultured separately in islet-packets. The device is see-through which gives the possibility of imaging the cells while performing functionality analysis such as OCR and insulin measurements. The islets could be lysed inside the device for further analysis of gene expressions, measurement of DNA methylation, and miRNA secretion by islets [49, 63].

Various microfluidic platforms have been designed using see-through materials in order to provide imaging opportunity of the cultured islets [64]. Integrating live-cell imaging technologies such as Raman imaging to the chip platform provide an excellent feature for non-destructive in situ imaging of islets in PoC system. Using this technology, one could visualize islet response to glucose and other stimulus as well as cell death stages, which all give better understanding of islet quality post-isolation [28•].

## Pancreas-on-a-Chip Application for Drug Development

In vitro studies for drug development mainly use cell culture protocols in which cells are grown on 2-dimensional (2D) monolayer in a well-defined culture medium. However, culturing cells in 2D is opposed to the cell micro-environment in living organisms, where all cells are in three-dimensional (3D) environment and have cell-cell and cell-extracellular matrix (ECM) interactions. ECM plays a critical role in determining the function and behavior of cells. In the context of the pancreas, interactions between the highly innervated and vascularized islets and the surrounding ECM are central for optimal insulin production and glucose responsiveness [65].

Animal models provide 3D cell micro-environment; however, the success rate of translating the results to human clinical trials have been noticeably low due to the major evolutionary differences between human and animal model. OoC technologies have a potential to mitigate several of the current limitations regarding the animal models and the in vitro studies in 2D systems by providing a standardized 3D culture system for human-relevant studies in a condition mimicking the cell micro-environment in vivo (Fig. 1).

Immunosuppressive medication which is an inevitable part of the islet transplantation could also get benefit from OoC technologies. OoC devices can provide a platform for testing the compatibility of the immunosuppressive regimen with immune system [66–69], as well as the effect of the immunosuppressive reagents on isolated islet function and viability [70]. OoC technologies have been used for studying the effect of immunosuppressive medications, tacrolimus, and cyclosporine A on T cell interactions and adhesion to endothelial adhesion molecules [71]. Rodrigues-Moncayo et al. developed a microfluidic chip with integrated biosensors that automatically measures interleukin-8 and tumor necrosis factor-alpha secretion from a consistent number of blood-derived monocytes, neutrophils, and THP-1 monocytes [66]. Such devices could be used for studying instant blood-mediated inflammatory reaction (IBMIR) and auto- and allo-immunity. They also give researchers an opportunity to investigate the effect of immunosuppressive reagents on insulin secretion in response to glucose [19, 72]. Future development of microfluidic platforms for islets together with compartments of immune systems and other tissue types especially liver tissue provide an opportunity to study the effect of immunosuppressive regimen on glucose metabolism and insulin secretion in a more standardized setting.

It should also be mentioned that in drug development efforts, it is desirable to perform direct measurements of drugs, drug metabolites, biomarkers of toxicity, etc. A common approach for such measurements is employing mass spectrometry (MS). The MS instrument allows analysis of small molecules (drugs, lipids, peptides) as well as larger biomolecules. MS enables both identification and quantification of compounds. The instrument allows for same-run measurements of large numbers of compounds, for example in metabolomics and proteomics. MS is often dependent on coupling with electrospray ionization (ESI) unit which allows biomolecules to enter the MS in gas phase (a requirement for MS analysis). In addition, compounds must often be separated prior to ESI-MS to secure ample sensitivity and secure identifications. This is typically performed using liquid chromatography (LC). Therefore, when mass spectrometry has been employed in bioanalysis/drug analysis, it is in reality a combination of these three techniques: LC-ESI-MS. However, LC-ESI-MS is as of today not commonly coupled with OoC technologies [73]. Common reasons are that online systems can be difficult to hyphenate due to incompatibilities (for example, 3D cell cultures can contain salts and proteins that can contaminate the LC-ESI-MS if a sample clean-up is not undertaken). In addition, the LC step can also dilute bio-fractions that may already be low in volume or analyst concentrations. Solutions to online coupling of OoC and LC-ESI-MS may lie in focus on online sample clean-up and reducing LC dilution. Regarding the former, one approach might be to incorporate electromembrane extraction, which is essentially an electrophoresis across an oil membrane. Initial efforts in combining EME and 3D models have recently been described and show promise for online action. Regarding the latter, drug analysis can potentially be undertaken using miniaturized LC columns, which are routinely used in, e.g., proteomics [74]. In any case, approaches to coupling OoC and mass spectrometry will be technically challenging but can strengthen the data output in drug development efforts.

## Generation of Multi-Organ-on-a-Chip for Islet Research

Organ-on-a-chip platform gives a possibility to combine and culture different cell types in separate culture compartments and connect them through microfluidic channels in order to mimic and study the cross-talk between two or more organs [75]. The concept is known as multi-organ-on-a-chip (MoC) or body-on-a-chip. However, each organ has its own specificity that needs to be considered in chip design in order to mimic the organ micro-environment. The more similar the physiology and property of organs are to each other, the easier and reliable the chip becomes to the native tissue. The Shuler research group is one of the pioneers in the field of MoC. They have developed MoC with multiple organ chambers called micro cell culture analog for co-culturing lung, liver, and tumor tissue to study the effect of anti-cancer medications on these tissue types [26•, 76, 77]. Imura and colleagues developed an MoC device for integrating breast cancer cells, small intestine, and liver [78] as well as integrating kidney tissue [79] for studying the absorption rate and therapeutic mechanisms of anti-cancer medications. There has been attempt of combining pancreatic islets and liver hepatocytes to investigate the physiological regulation of circulation glucose by the biological source of insulin produced by pancreatic islets in the system [52•]. This system represents a 15 day co-culture of islet and liver spheroids in which functional islets responded to glucose load to the system and produced insulin. Secreted insulin promoted glucose uptake by liver spheroids, and as the glucose concentration fell, insulin secretion by islets also decreased, demonstrating a feedback loop between liver and islets [52•]. This platform could be a useful technique for studying the effect of specific medications including diabetic or immunosuppressive medications on transplanted islets and the effect on islet-liver communication.

Pancreatic islets are heavily vascularized in vivo with fenestrated endothelial cells (ECs) to facilitate blood glucose sensing and endocrine hormone secretion [37]. Islets possess a strong metabolic regulation of their blood perfusion. Of particular importance is adenosine and ATP/ADP. Basal and stimulated blood flows are modified by local endothelial mediators such as nitric oxide, the sympathetic/parasympathetic innervation as well as gastrointestinal hormones [80]. The close proximity of insulin-secreting beta cells and ECs also play a critical role in modulating the proliferation and survival of both cell types [37]. During islet isolation procedure, islets lose their vascularized capillary, which consequently make them prone to hypoxia and could affect their endocrinal functions. Therefore, generation of vasculature network together with islets on MoC platform provides a possibility of mimicking the physiological interactions of islets with this network and other organs in MoC. Endothelium is not a planer surface but rather has tubular structures with various sizes, which could impose challenges in engineering of the microfluidic chips [81]. However, advances in soft lithography allow us to construct transparent microfluidic devices with micro-channels in which endothelial cells can be coated the inner surface of the channel to create blood vessel network [82].

For modeling blood vessels in vitro, human umbilical vein endothelial cells (HUVECs) are the popular choice due to their accessibility and vast knowledge regarding their expressing specific markers. There have been attempts in generating vasculature model on MoC together with different tissue types. Spheroids generated from lung fibroblast were cultured in a microfluidic chip with endothelial cells in the micro-channel of the chip. This study has been shown successful angiogenesis of the endothelial cells toward lung tissue [83]. Stevens et al. has also created a model for liver vasculature using primary human hepatocytes and HUVEC cells. In this mode, liver organoids were combined with geometrically patterned human endothelial cells in a hydrogel that has the possibility to be implanted into a microfluidic device [84] (Fig. 1).

Blood vessels have the capability to sprout in vitro on a chip platform as shown by Duinen et al. [85]. This capability could be exploited in a two- or three-compartment chip format in which HUVEC cells or iPS-derived vascular endothelial cells are in one compartment, while parenchymal islet cells are in a second chamber separated by columns or a ridge. A third chamber may serve as a recipient for the vascular endothelial cells such as veins would do in the body. In this setting, endothelial cells may sprout into the target tissue and supply it with nutrition, oxygen, and physiological molecules such as glucose, hormones, and metabolites in a controlled way, and exit the molecules that are produced by islets into the venous system from where they can be measured. Indeed, Rambol et al. have recently presented a microfluidic system for islet vascularization [86] in which HUVECs were supported by mesenchymal stem cells (MSC) and formed perfusable endothelialized networks in a fibrin gel. Subsequent incorporation of isolated rat islets demonstrated that islets recruit local microvasculature in the device.

## Pancreas-on-a-Chip Application for Generation and Evaluation of Pluripotent Stem Cell- or Non-endocrine Pancreatic Cell-Derived Beta-like Cells

Despite the impressive advances in the field of human islet isolation, scarcity of suitable donors for islet isolation procedure affects the amount of islets that can be used for research and for clinical islet transplantation. To target the shortage of pancreatic islets, alternative sources of cells including differentiation of stem cells and progenitor cells into insulin-producing beta cells or transdifferentiated non-endocrine cells to beta-like cells have been investigated over the years. Differentiation of ESCs or iPSCs toward specialized cell types is based on an in vitro recapitulation of the sequence of inductive signals that cells are exposed to during embryogenesis at different stages of tissue/organ development. From the first published stage-to-stage protocol in which human pluripotent stem cells were guided through developmental stages of the pancreas resulting in immature multi-hormone-expressing endocrine cells [1, 2, 9, 87] until now, the differentiation protocols were considerably improved in reaching partially functional beta-like cells [88–90]. As a result, for the first time, some of the ES-derived endocrine cells exhibited delayed and less prominent glucose-stimulated insulin secretion compared to the human islets [88], and these cells were able to reverse hyperglycemia in chemically induced diabetic mice [3].

OoC platforms can be used as a 3D culture method offering new alleys as highlighted by the importance of cell-to-matrix and cell-to-cell interactions as well as mechanical cues for the development of cell fates during the development of islets [91–93]. Re-aggregation or encapsulation of pancreatic progenitors or immature beta-like cells have been shown as one of the essential steps toward improving the maturation and functionality of stem cell-derived beta-like cells [94–96]. In particular, Velazco-Cruz et al. has reported improvement in glucose responsiveness of stem cell-derived beta-like cells as these cells were able to have both first and second phase of insulin release by generation of uniform-sized cell clusters [95]. A multi-layer OoC platform has been used for aggregation and differentiation of islet organoids containing alpha and beta cells. These cells expressed endocrine markers and were responsive to glucose-stimulated insulin secretion test [50].

Pancreatic islet survival and function are dependent on islet microvasculature network. During pancreatic development, endothelial, mesenchymal, and neuronal cells form the micro-environment required for proper organogenesis [97–99]. Moreover, within the endocrine part of the pancreatic tissue, insulin secretion by beta cells is affected by the paracrine signals produced by non-beta cell fraction of endocrine tissue [100–102]. Therefore, absence of micro-vasculature environment compromises functionality of stem cell-derived beta-like cells. Engineering organoids containing human islet and amniotic epithelial cells have been reported to improve organoid function and engraftment in type 1 diabetes mouse model [103]. These types of studies are yet to be done for generating uniform stem cell-derived organoids together with other cell types such as endothelial, neuronal, and mesenchymal cells in order to enhance function and vascularization of organoids. Combination of organoid engineering, 3D culture approaches, and microfluidic platforms could serve as a toolbox for recapitulating the precise spatio-temporal niche signals during pancreatic development as well as copying the dynamics of blood flow and mechanical forces during islet formation and functionality as a mature organ. Advancing of technologies could also help in deciphering of mechanisms coordinating endocrine cell clustering and islet formation.

Reprogramming pancreatic non-endocrine acinar or ductal cells to insulin-producing beta-like cells could be another approach for reducing the demand for beta cells. These cells could have extreme plasticity with a strong potential to be reprogrammed to insulin-producing beta-like cells [104, 105]. Although acinar cells have been shown to have a potential to generate insulin-positive cells, the state of acinar to ductal cell dedifferentiation was observed before endocrine reprogramming, which could suggests higher plasticity of ductal cells [106–109]. In addition, severe damage to pancreatic tissue such as acinar ablation and partial duct ligation (PDL) has been shown to induce differentiation toward endocrine lineages including beta cells. This evidence suggests the existence of a pancreatic progenitor pool within the ductal tree in the pancreas tissue [110, 111]. Most of the current differentiation protocols are conducted in a 2D culture, followed by suspension aggregate cultures or cultures at an air-liquid interface [14]. Although, this approach works for stem cells during the early stages of differentiation protocol, it is worth mentioning that expansion and transdifferentiation of non-endocrine pancreatic cells to beta-like cells requires 3D culture system. Transdifferentiation and expansion of these cells in 2D culture system could hamper the differentiation process by transition of non-endocrine cells to mesenchymal cell-like phenotype during the culturing process [112, 113]. Culturing duct progenitor cells in a matrigel-based 3D culture system has been shown to facilitate the differentiation of these cells to insulin-producing cells [114]. Hence, reprogramming of pancreatic non-endocrine cells to insulin-producing cells could also get benefit from OoC technologies.

## Challenges and Outlook

PoC technology has recently become more accessible and robust. Although the field is still in its infancy, there have been huge efforts from the field of the microfluidic engineering to develop new and functional PoC platforms.

Materials for PoC fabrication need to support both the engineering of the device and cell survival. Most of the PoC devices are made of PDMS. The material is highly deformable, which makes it applicable to replicate structures at high resolution. It is also biocompatible, oxygen permeable, and optically transparent. All these factors make this material suitable for biological studies. However, the flexibility and gas exchange property of PDMS lead to evaporation of culture media. This is mainly problematic in PoC applications due to having small volume of the cell culture medium in these systems [115]. In addition, PDMS has been shown to absorb lipophilic compounds such as steroid hormones and various drugs affecting the islet analysis [116, 117]. New materials are under development; however, they have not been widely used, and it could be beneficial to use various materials based on the application purpose.

Despite all the improvements in the field of engineering to create PoC platforms, designed chip systems need to meet specific biological requirements in order to mimic organs in multicellular organisms. Amount of cells/tissue on chip, the ratio of each tissue types in MoC, and flow rate are some of the factors that could disturb the application of PoC models. The main biological issue is related to the source of cells that are introduced to PoC systems. Current human PoC formats are based on isolated islets from cadaver donors that are either used directly post-isolation or re-aggregated to a standard-sized format. In fact, human islets are limited due to their cost and few available donors. Therefore, human stem cell-derived beta-like cells are promising sources. However, in vitro differentiated beta-like cells have not reached enough metabolic maturity to be suitable for a truly physiological on-chip format. Moreover, isolated islets lack the exocrine part of the pancreas that surrounds them, in particular the ductal epithelium from which they have emerged during development and to which they are still attached [118–120]. Isolated islets do not contain components of the immune system that may surveillance and interact with them [120]. The same is true for in vitro-differentiated beta-like cells. Without those elements, PoCs will per se only be able to copy a limited set of physiological functions. This may not be a problem for pre-transplantation testing as the islets will be vascularized and innervated through the host after transplantation. However, for ex vivo islet models that shall be functioning as close as possible to in vivo, on-chip vascularization and innervation may be an interesting option.

Previously developed microfluidic models have used cell culture medium to provide various metabolites, nutrients, and oxygen for cells. However, different tissue types require different cell culture medium, and this issue becomes more serious when attempting culture of various tissue types in one platform. Therefore, finding a universal culture medium is an extremely urgent matter.

Complicated handling procedure of PoC devices greatly affects high-throughput, usability, and standardization of these devices. This imposes a major barrier to wider practical application of this technology. Most of the designed PoC platforms require many sample collections. This could disturb operation of the chip and also might change the concentration of various metabolites during the sampling procedure. Generating in situ monitoring systems and using sensors to monitor the situation of the islets in an automated manner could be a way to have standardized and more reliable systems.

PoC platform generation for islet research is a rapid developing area. A number of examples are cited in this review, demonstrating the advantages of having functional PoC systems to evaluate islet quality post-isolation. Developing PoCs that could better reflect the complexity of islets as a single tissue type or combined with other types of tissue on MoCs could create a platform for diabetes disease modeling and drug testing especially diabetic medications and immunosuppressive regimen which all could be beneficial for improving the outcome of islet transplantation procedure.

## Abbreviations

ESC: Embryonic stem cell
IBMIR: Instant blood-mediated inflammatory reaction
iPSC: Induced pluripotent stem cell
T1D: Type 1 diabetes
OoC: Organ-on-a-chip
MoC: Multi-organ-on-a-chip
PoC: Pancreas-on-a-chip
ECM: Extracellular matrix
MS: Mass spectrometry
EC: Endothelial cells
HUVEC: Human umbilical vein endothelial cells
